# Surveying Alcohol Outlet Density in Four Neighborhoods of Beirut Lebanon: Implications for Future Research and National Policy

**DOI:** 10.3390/ijerph15092006

**Published:** 2018-09-14

**Authors:** Rima Nakkash, Lilian A. Ghandour, Sirine Anouti, Jessika Nicolas, Ali Chalak, Nasser Yassin, Rima Afifi

**Affiliations:** 1 Department of Health Promotion and Community Health, Faculty of Health Sciences, American University of Beirut, Beirut 1107-2020, Lebanon; rn06@aub.edu.lb (R.N.); rema-afifi@uiowa.edu (R.A.); 2 Department of Epidemiology and Population Health, Faculty of Health Sciences, American University of Beirut, Beirut 1107-2020, Lebanon; sa228@aub.edu.lb; 3 Department of Geology, Faculty of Arts and Sciences, American University of Beirut, Beirut 1107-2020, Lebanon; jtn04@mail.aub.edu; 4 Department of Agriculture, Faculty of Agricultural and Food Sciences, American University of Beirut, Beirut 1107-2020, Lebanon; ac22@aub.edu.lb; 5 Department of Health Management and Policy, Faculty of Health Sciences, American University of Beirut, Beirut 1107-2020, Lebanon; ny05@aub.edu.lb; 6 Department of Community and Behavioral Health, College of Public Health, University of Iowa, Iowa City, IA 52242, USA

**Keywords:** GIS (geographic information systems), alcohol, youth, alcohol density, WHO best buys, alcohol availability, Lebanon, Beirut

## Abstract

Underage drinking among youth in Lebanon is increasing. Regulating availability is one of the best buy policies recommended by the World Health Organization. To quantitatively document the current status of alcohol availability to youth in Lebanon, we used GPS technology to survey alcohol outlet density in four highly populated neighborhoods in Beirut, Lebanon, and to estimate their proximity to educational institutions. The density of alcohol outlets ranged from 18.30 to 80.95 per km^2^ (average of 39.6 alcohol outlets/km^2^). The highest number of total alcohol outlets was in the “Hamra & Jamiaa” area, which includes one of the largest private universities in the country. Thirteen out of 109 (12%) alcohol outlets (on and off-premise) were located less 100 m away from educational institutions, in violation of the current licensing law. None of the off-premise and the majority (94%) of on-premise alcohol outlets displayed the “no sale for <18” sign. Findings were indicative of an environment conducive to increased access and availability of alcohol among youth in Lebanon probably attributed to the prevailing weak alcohol policies and their enforcement. Systematic collection and reporting of alcohol outlet densities is critical to understand the alcogenic environment and guide local harm reduction policies.

## 1. Introduction

Alcohol has been identified as a leading risk factor for death and disability among young people aged 15–24 years worldwide [1]. Early onset of alcohol drinking has been consistently linked to poor outcomes among youth, including poor academic achievement [2], suicide ideation [3], high-risk sexual behaviors [4,5], and alcohol-related road traffic accidents [6]. Early initiation of alcohol drinking has been also linked to a higher likelihood of developing alcohol dependence later in life [7,8,9], and to various non-communicable diseases in adulthood [10]. Rising consumption among youth can be partially explained by the the heavy targetting of youth through advertising, especially in low-middle income countries where alcohol markets are emerging [11,12,13].

A range of evidence-based ‘best-buy’ policies have been recommended as effective solutions to reduce alcohol-related harms [14,15]. Regulating physical availability of alcohol is one strategy and includes policies that restrict alcohol outlet density (AOD), defined as the number of outlets where alcohol is sold (such as bars, restaurants, and liquor stores) in a given geographic area or per population [11,16,17,18]. Evidence indicates that high AOD is positively associated with excessive alcohol drinking and related harms [17]. In addition, high AOD contributes to the ‘alcogenic’ environment of a community; greater availability of alcohol reduces the “convenience costs” of obtaining it increasing average consumption within a society [19], and normalizing alcohol consumption [20]. In particular, high AOD contributes to youth alcohol drinking by enabling youth access [21,22,23], underage drinking [24,25,26], excessive alcohol consumption [21,27,28], drunk-driving [29] and other alcohol related harms [18,29,30]. In addition, evidence has shown that increases or decreases in AOD have resulted in direct changes in alcohol consumption and related-harms [28,31,32].

Alcohol outlets are categorized into two groups: off-premise (i.e., places that sell alcohol beverages that are meant for consumption elsewhere, such as supermarkets, liquor, and grocery/convenience stores) and on-premise (i.e., places that sell alcohol beverages for on-site consumption such as restaurants, coffee shops, bars and pubs). AOD restrictions may involve either off-premise or on-premise outlets, or both. Both on- and off-premise AOD restrictions have been reported to reduce alcohol consumption and alcohol-related harms [18], in line with the World Health Organization (WHO) best buy policies [22,32,33]. The use of regulatory authority, e.g., zoning and licensing, has been recommended as a strategy to reduce AOD [18,26]. Access to alcohol among youth may also be reduced by banning sale of alcohol in outlets situated near schools, universities, and hospitals [18,34]. Such measures are expected to increase the distances traveled to obtain alcohol, raise prices through reducing competition and decrease exposure to points of purchase [16,26].

Despite this plethora of evidence, countries worldwide have varied in the extent to which they have regarded alcohol related policies and their implementation as a national health priority. This policy laxness is exacerbated by active marketing of alcohol companies to adolescents and young adults, particularly in the less developed world [13]. Many countries have adopted or are in the process of developing a national alcohol control policy in response to the WHO Global strategy to reduce the harmful use of alcohol [13]. Lebanon is an example of a small country in the Arab world, which is characterized by weak, outdated, and almost non-existent alcohol-related policies [35], creating a lax regulatory environment. Many alcohol beverages are inexpensive, illegal sale to minors is common, and advertising/marketing of alcohol products is unregulated. Available evidence shows that alcohol consumption among adolescents and young adults in Lebanon is a rising public health concern. Past-month drinking among 7th to 9th graders increased by 40% between 2005 and 2011, with 85 percent having had their first drink before the age of 14 [36]. Youth in the country also drink more frequently than occasionally; a high school survey showed that 40% of the past-year drinkers reported consuming alcohol at least 1–2 days per week [37]. Similarly, among university students, about half of the past-year drinkers reported consuming alcohol 1–2 days per week [38]. Perceived easy access to alcohol is a clear driver of youth drinking choices in the country [36]. The current laws governing the physical availability of alcohol are related to licensing of on-premise outlets (e.g., restaurants, cafes, bars, or pubs) including restrictions on the licensing of new bars confining them to non-residential areas (i.e., touristic and commercial) with a minimum distance of 50 m separating bars (Decision no. 3210, issued in 1974). Nonetheless, in the capital Beirut, the lack of urban planning and overlap between residential and non-residential areas renders these regulations largely inapplicable. Also, there are no policies that regulate the density of off-premise alcohol outlets such as supermarkets, liquor stores, convenience stores, gas stations and/or grocery stores [35].

As part of a larger research program aiming to generate data to inform a national alcohol harm reduction policy, we conducted a study to examine the AOD in four highly populated neighborhoods of Beirut district (the largest and capital city of Lebanon), using Global Positioning Systems/Geographic Information System (GPS/GIS) technology, similar to previous research [39,40,41]. Specifically, we: (1) assessed the AOD by type of premise (on- and off-) and beverage type; and (2) created geographic reference points corresponding to each type of alcohol outlet that was identified in order to track its distance and adjacency (proximity) to educational institutions. The findings are discussed and benchmarked vis-à-vis other global cities where possible. The paper also discusses the ramifications on risks of alcohol-related harms among youth in Lebanon and similar contexts.

## 2. Materials and Methods

### 2.1. Field Work

**Figure 1:**
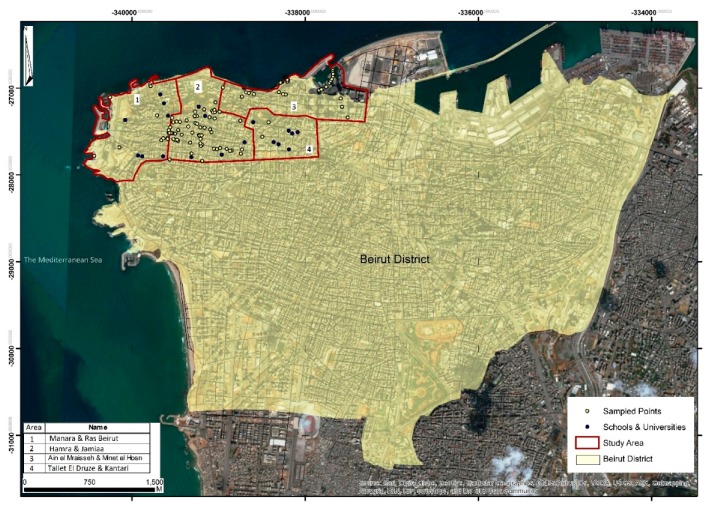
Beirut district with the surveyed study area outlined in red.

Data collection was supervised by an ArcGIS software specialist, who recruited, trained and supervised four fieldwork-experienced graduate students enrolled at the American University of Beirut (AUB) at the time of the study. Prior to initiating the survey, two areas in Beirut district distant from the study areas were selected for pilot-testing the survey form and fieldwork. The trained fieldworkers surveyed each area street by street to identify the number and location of all on-/off-premise outlets. The pilot phase was held over four days, followed by a debriefing meeting held with the research team to discuss findings and make all necessary amendments to the survey. The study did not require formal approval of the Institutional Review Board since it did not involve human subjects. Data collection for the actual study was initiated on 3rd June and completed on 2nd July 2014 across four purposively selected areas in Beirut, representing highly populated and residential areas of the capital city of Lebanon: (1) “Manara & Ras Beirut”; (2) “Hamra & Jamiaa”; (3) “Ain el Mraisseh/Minet el Hosn”; and (4) “Tallet El Druze & Kantari” (Figure 1). The geographical boundaries that were used in the maps were extracted from the civil maps of the Lebanese government.

Each data collector used a hand-held eTrex ^®^10 GPS locator (GARMIN, Olathe, KS, USA and supplied by the Geology department at AUB) for high accuracy positioning purposes. Geographic points for each outlet were identified on the handheld GPS using four to five satellites with a 5 to 7 m precision. The fieldworkers enumerated and mapped all the outlets in the area and noted on the observation form specifically: (1) location/coordinates of the outlet; (2) type of outlet (on- and off-premise); (3) whether or not the outlet sold alcohol; (4) the type(s) of alcoholic beverages sold; (5) opening/closing time; (6) whether or not the off-premise alcohol outlet has a sign of “no sale to <18”; (7) advertisement/promotions of alcohol; and (8) location/placement of alcohol products inside the outlet. Data collectors completed the survey form after observing the outlet and/or clarifying information with the shop owners when necessary. The collected locations were coupled with the information filled by the data collector on the survey form. In order to ensure the quality of the data, the ArcGIS specialist shadowed the fieldworkers in the field, and cross-checked the data obtained using Google Earth. No discrepancies were found, yet the process also allowed the supervisor to monitor the fieldworkers’ daily paths.

### 2.2. Data Analysis

Upon the completion of fieldwork, the data were shared with the ArcGIS specialist for pre-proofing using available databases and producing detailed maps. The location of educational institutions was also identified on the map in two ways: location of schools within the areas surveyed was noted by the field workers during data collection, and the ArcGIS specialist cross-checked the data against a database with all the available schools and universities within the study areas.

The GIS data collected was displayed and analyzed using ArcGIS version 9.1, 9.2 (ESRI, Redlands, CA, USA), a geographic information system software platform. The geocoding tools helped create a geo-database with a relative address locator for each outlet identified during field work; then, depending on the locations of the data being analyzed, the online mapping tools produced analytical and comprehensive maps. ArcGIS Spatial Analyst tools were used to: (1) analyze patterns and map clusters in both stand-alone and relative modes; and (2) generate the relative distance and cluster density comparison maps. Cluster analysis was done to compare the point distribution to the designated area.

AOD was calculated using the total number of outlets and the geographic surface area in km^2^ of each of the four surveyed areas in Beirut district. We calculated density for all outlets and for on- and off-premise alcohol outlets separately, and then analyzed the distribution by type of outlet, type of alcoholic drinks they serve/sell, as well as their proximity to schools & universities.

## 3. Results

### 3.1. Density of Outlets

The most common alcohol outlets in the surveyed area were restaurants (55%). Out of the 221 total outlets that were mapped in these areas, almost half (*n* = 109, 49%) were alcohol-selling outlets, the focus of our paper (i.e., restaurants or grocery stores with no permit to serve alcohol are non-alcohol-selling outlets that have been excluded from this analysis). Of the 109 alcohol-selling outlets, 83 (76%) were on-premise. Table 1 presents the characteristics of the surveyed on-premise and off-premise alcohol outlets in the four neighborhoods surveyed. The ratio between on-premise and off-premise alcohol outlets was about three to one. The most common types of on- premise and off-premise alcohol outlets were restaurants (*n* = 60, 72.29%) and mini-markets/small grocery stores (*n* = 20, 77%), respectively.

**Figure 2:**
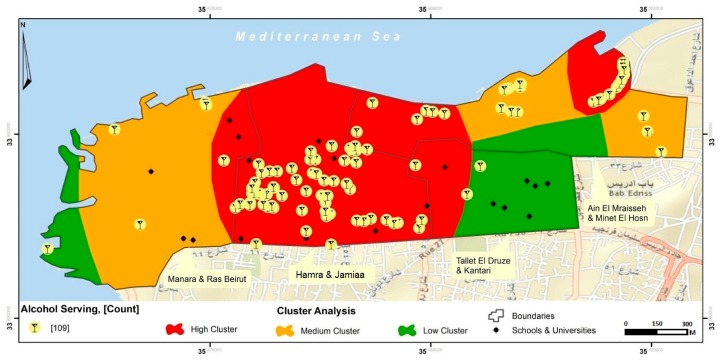
Raster analysis showing spatial distribution of alcohol outlets.

Beer and wine were the two most available types of drinks in on-premise alcohol outlets whereas alcoholic energy drinks and beer were the two most widely available in off-premise alcohol outlets (Table 1). A third of on-premise outlets and approximately 70% of off-premise outlets offered all types of alcoholic beverages (Table 1; Figure 2). All of the off-premise outlets (*n* = 26) and the vast majority of on-premise outlets (*n* = 77, ~93%) did not display a “no sale for <18” sign.

**Figure 3:**
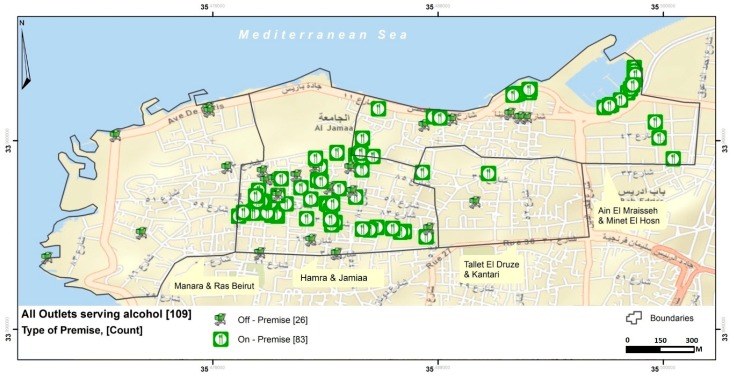
On-premise and off-premise alcohol outlets in Beirut district.

The density of alcohol outlets ranged from 18.30 to 80.95 per km^2^ (Table 2; Figure 3) depending on neighborhood. The neighborhood with the highest number of total alcohol outlets was “Hamra & Jamiaa”, which includes one of the largest private universities in the country. A higher density of on-premise compared to off-premise alcohol outlets can be observed for this area (Table 2; Figure 2).

### 3.2. Distances from Educational Institutions

**Figure 4:**
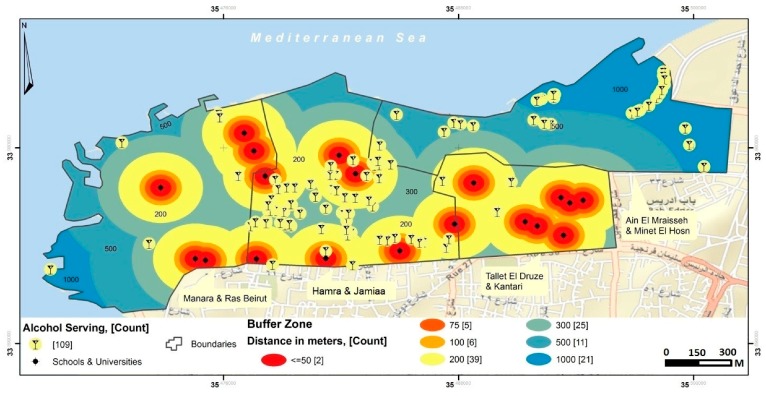
Distance from schools and universities to alcohol outlets.

There were 19 educational institutions (seven universities and 12 schools) in the surveyed area. The nearest alcohol outlet to an educational institution was an off-premise outlet situated 43 m away in the “Hamra & Jamiaa” neighborhood. When the analysis was stratified by the type of educational institution closest to the individual outlets, the shortest distance to the nearest university was an off-premise situated at 43 m and that to the nearest school was also an off-premise situated at 52 m away; both located in “Hamra & Jamiaa” neighborhood (Table 3; Figure 4).

Using different radii to create buffer zones (Figure 4), the proportion of alcohol outlets within 300 m of an educational institution reached 70% (i.e., 77 out of 109) (Table 1). Moreover, these alcohol outlets were clustered closer to schools than universities (66% vs. 60%) (Table 3; Figure 4). Although a distance of 300 m away is commonly used in research on the influence of environmental factors on health-related behaviors among youth and older population [42,43], Lebanese law states that alcohol outlets must be at least 100 m away from schools. We found nine out of 109 (8%) alcohol outlets (on and off-premise) located at a distance of less than 100 m away from schools of which more than two-thirds (67% or 6 out of 9) are on-premise alcohol outlets (Table 3). Also, out of these 9 “law-violating outlets”, 2 (15%) sell beer, 1 (33%) sell alcohol energy mixes, and 10 (77%) sell more than one type of alcohol; and they were all located in the “Hamra & Jamiaa” neighborhood. None displayed the “no sale for <18” sign (Table 4). We also compared outlets that are close to schools vs. those that are more distant, in terms of their practices and found no marked or statistically significant differences (Table 4).

## 4. Discussion

Results from the surveillance of the four populated neighborhoods in Beirut district clearly indicate an alcogenic environment [11,44]. We found an average density of 39.6 alcohol outlets/km^2^, which exceeds that reported for other cities, such as Toronto (Canada, 2.9 per km^2^) [45], New York City (USA, 23.55 per km^2^ in 59 community districts) [46] or Sao Paolo (Brazil, 29 per km^2^) [47]. Clearly, all these cities vary in size, context, commercial activity, culture as well as local alcohol policies, but they are all big cities with densely populated residential areas as in Beirut. Comparability of our findings to other studies conducted in a similar culture would be especially hard given the scarcity of existing literature exploring this topic in the Arab countries with similar contexts [35]. The relatively high observed AOD may be attributed to the country’s prevailing weak alcohol policies and/or policy enforcement, favoring an environment characterized by easy alcohol access and availability [35]. Our study also illustrated the high AOD particularly around local educational establishments. Other studies have reported variable AOD around educational establishments, with some studies reporting generally high AOD around university and college campuses and linking it to increased alcohol consumption and/or violence [48,49,50].

International evidence clearly indicates that alcohol consumption and related harms increase as AOD increases in general and among youth, and supports limiting AOD to reduce alcohol harm at the population level [18,33,51,52]. Research, however, falls short on recommending density thresholds because of the incomparability of the methods used, and differences in geographic and community characteristics [53]. Consensus does exist, however, on the critical importance of local information, sound theoretical framework and well-designed research for understanding the specificities of the alcogenic environment in any particular context [19]. According to the Centers for Disease Control and Prevention (CDC) comprehensive guide for measuring AOD, it is essential that public health agencies monitor high AOD through collecting and reporting AOD and “clustering” to the local and municipal levels to help inform evidence-based strategies for preventing excessive alcohol use and related harms [54].

Considering the outdated alcohol availability laws in the country, our data shows a clear violation of the local policy relating to distance from schools. Our GPS surveillance detected a total of 9 alcohol outlets (on and off-premise) at a distance of less than 100 m away from schools of which 77% sell more than one type of alcoholic beverages and importantly none displayed the “no sale for <18 years” sign. Furthermore, alcoholic beverages of any type can be found at a minimum distance of approximately 58 m away from a school. This is a clear violation of Decision number 208 issued in 2010 that dictates the denial of any licensing of alcohol outlets if the entrance of the outlet is within 100 m of a school. The latter does not encompass universities; in fact, the regulations regarding the licensing of places for sale and consumption of alcohol do not address universities at all. The same decision includes a stipulation allowing “seasonal touristic institutions/enterprises” to offer spirits drinks, conditional on their conforming with the laws and regulations in force, and their location at a distance of more than 20 m from schools’, sanctuaries, and hospitals.

Another WHO recommendation is to establish an appropriate minimum age for purchase or consumption of alcoholic beverages and other policies in order to raise barriers against sales to, and consumption of alcoholic beverages by, adolescents. Our results indicate that an overwhelming majority of the alcohol outlets in Beirut district do not display signs indicating “no sale for <18”. The local law on minimum legal drinking age was last revised in 1993 and imposes very minimal sanctions and fines (10,000 to 20,000 LBP equivalent to 6.5 to 13 USD) on owners and employees of bars/pubs or other similar places that are open to the public, in cases where they offer spirit drinks to drunk persons, or to minors under age of 18 years or in cases where they put a person in a drunken state. The law only addresses on-premise outlets (pubs/bars), and says nothing about sales to minors in off-premise outlets [35].

Our findings clearly indicate that young people living in the capital city of Lebanon (an economic, diplomatic, and tourist hub), can easily access alcohol from a variety of commercial sources, corroborating earlier survey data where approximately 1/4 of middle school students reported “buying (alcohol) from store/shop/street vendor” [36].

In general, findings from this research suggest a high AOD conducive to increased drinking among youth with its potential concomitant negative consequences. AOD was measured here through density per km^2^. Previous research has used this as well as alternate measures of AOD such as alcohol density per capita, which we were unable to calculate because of the lack of updated census data at the national level. This limited our ability to further compare our results with other studies. Also, certain establishments including beach resorts and gas stations were not mapped, which may contribute to an underestimation of the alcohol outlet density. We acknowledge that the four neighborhoods we selected may not be representative of all of Beirut, nor of Lebanon, but we selected these areas with intent to begin to quantify and understand actual physical availability of alcohol in such a lax policy environment. This study provides a baseline assessment of the situation at one point in time in 2014. The generated data serve as an evidence-based case presentation aiming at sensitizing the regulatory authority to limit alcohol density in an effort to regulate availability of alcohol among youth [17,55]. Follow-up studies to further understand changes in AOD and patterns of alcohol sales are warranted to better understand the context, as well as inform and monitor alcohol control policy. We also acknowledge that AOD is only a measure of potential access, not actual behavior. Future research should explore the link between GPS surveillance data and data on alcohol consumption, and related harms such as alcohol-related fights or drunk-driving cases in the neighborhoods with high outlet densities. This will require sustainable surveillance mechanisms by local authorities as well as access of researchers to such information to strengthen advocacy efforts and ensure the implementation of evidence-based regulatory measures.

## 5. Conclusions

Our work should raise attention to the need for further studies to understand if and how this documented alcogenic environment that pervades these neighborhoods in Beirut, specifically around universities, might be leading to higher alcohol consumptin or other alcohol related harm among this population. Changes in land and zoning rules within the city of Beirut—such as establishing alcohol-free zones for schools and universities might be one potential recommendation for the future [43]. The impact of these policies on alcohol availability and the broader agenda of alcohol control will require active and sustainable public health surveillance and a collaboration between state and local public health departments, community coalitions and other partners [16]. Policies such as minimum legal drinking age and pricing also contribute to higher youth alcohol consumption [56]. Advocacy and coalition building around alcohol control is a priority to mitigate health consequences of the current status quo and protect wellbeing of young people in Beirut. These efforts can serve as a model for expansion of this work nationally.

## Figures and Tables

**Table 1:** Characteristics of the surveyed on-premise and off-premise alcohol outlets in the four neighborhoods of Beirut district.

Outlet Characteristics	All (*n* = 109)	On-Premise *n* = 83 (76%)	Off-Premise *n* = 26 (24%)
**Type of outlet *n* (%)**			
Large market/store or Supermarket	2 (2%)	-	2 (8%)
Mini-market/small grocery store	20 (18%)	-	20 (77%)
Convenience stores (gas station)	2 (2%)	-	2 (8%)
Liquor stores	2 (2%)	-	2 (8%)
Restaurants	60 (55%)	60 (72%)	-
Pubs/Bars	23 (21%)	23 (28%)	-
**Type of drinks sold *n* (%)**			
Spirits (whiskey, Arak, rum, tequila, vodka, et al.)	78 (72%)	57 (37%)	44 (68%)
Beer	103 (95%)	80 (51%)	23 (35%)
Wine	95 (87%)	75 (48%)	20 (31%)
Mixed energy alcoholic drinks	48 (44%)	24 (15%)	24 (37%)
More than one type	96 (88%)	73 (88%)	23 (88%)
All types	42 (39%)	24 (29%)	18 (69%)
**Proximity to educational institutions in meters *n* (%)**			
≤100	13 (12%)	7 (8%)	6 (23%)
≤200	52 (48%)	37 (71%)	15 (29%)
≤300	77 (70%)	59 (77%)	18 (23%)
**Display “No alcohol sale for <18” sign**			
Yes	6 (5.5%)	6 (7%)	0
No	103 (94.5%)	77 (93%)	26 (100%)

**Table 2:** Number and density of alcohol outlets by area surveyed.

Neighborhood	Area in km^2^	Number of Outlets	Number of Alcohol Outlets			Outlet Density (Number of outlets/km^2^)		
			All	On-Premise	Off-Premise	All	On-Premise	Off-Premise
Area 1 Manara & Ras Beirut	0.89	26	6	0	6	29.35	0	6.74
Area 2 Hamra & Jamiaa	0.86	144	68	55	13	80.95	63.95	15.11
Area 3 Ain ElMraisseh & Minet El Hosn	0.64	44	32	26	6	50.37	40.62	9.37
Area 4 Tallet El druze & Kantari	0.38	7	3	2	1	18.30	5.26	2.63
Total	2.75	221	109	83	26	39.63	30.18	9.45

**Table 3:** Distance between alcohol outlets and educational institutions.

Proximity of Alcohol Outlets to Schools and Universities	All Educational Institutions	
	Schools (*n* = 12)	Universities (*n* = 7)
**The shortest distance in meters to an educational institution by outlet type**		
On premise alcohol outlet	74.2	71.1
Off premise alcohol outlet	51.7	42.6
**The shortest distance in meters to an educational institution by area**		
Area 1 Ras Beirut & Manara	127.37	146.9
Area 2 Hamra & Jamiaa	51.7	42.6
Area 2 Ain Al Mraisseh & Minet El Hosn	174.6	199.5
Area 4 Tallet Jounblat & Kantari	143.4	148.8
**Number of outlets […….] meters away from educational institutions *n* (%)**		
≤75	3 (3%)	4 (4%)
≤100	9 (8%)	6 (6%)
≤200	37 (34%)	32 (29%)
≤300	72 (66%)	66 (61%)

**Table 4:** A comparison between the outlets that are close to schools vs. those that are more distant to schools.

Outlet Characteristics	Outlets ≤100 m Away from Schools *n* (%)	Outlets >100 m Away from Schools *n* (%)	*p*-Value
**Type of drinks sold**			0.296
Spirits	0	1 (1%)	
Beer	2 (15%)	5 (5%)	
Wine	0	2 (2%)	
Mixed energy alcoholic drinks	1 (33%)	2 (2%)	
More than one type	10 (77%)	86 (90%)	
**Type of outlet**			0.076
On-premise	6 (46%)	20 (21%)	
Off-premise	7 (54%)	76 (79%)	
**Display “No sale for <18’ sign”**			
Yes	0	6 (6%)	1.000
No	13 (100%)	90 (94%)	
